# Study of the Behavior of Structural Materials Treated with Bioconsolidant

**DOI:** 10.3390/ma14185369

**Published:** 2021-09-17

**Authors:** Yolanda Spairani, Arianna Cisternino, Dora Foti, Michela Lerna, Salvador Ivorra

**Affiliations:** 1 Department of Architectural Constructions, University of Alicante, San Vicente Del Raspeig, 03080 Alicante, Spain; yolanda.spairani@ua.es; 2 Department of Civil Engineering Sciences and Architecture, Polytechnic University of Bari, Via Orabona 4, 70125 Bari, Italy; arianna.cisternino@libero.it (A.C.); michela.lerna@poliba.it (M.L.); 3 Department of Civil Engineering, University of Alicante, San Vicente Del Raspeig, 03080 Alicante, Spain; sivorra@ua.es

**Keywords:** bioconsolidation, experimental campaign, non-destructive tests, mechanical tests, microstructural analysis

## Abstract

In this article, the effectiveness of the bioconsolidation technique applied to degraded structural materials is illustrated as a new method of consolidation and conservation of the existing building heritage in a less invasive way. Satisfactory results have been obtained by an experimental campaign carried out through non-destructive diagnostic tests, static destructive mechanical tests, and microstructural analyses on a series of natural stone material specimens and artificial stone materials before and after the use of bioconsolidants. The consolidated specimens have been tested after three to four weeks after the application of the M3P nutritional solution on each specimen. The effect on the microstructure of this technique has also been observed using scanning electron microscope and optical photomicrograph, the formation of new calcium carbonate crystals promoting the structural consolidation of the materials under examination was observed in all the specimens analyzed.

## 1. Introduction

The decay of the existing building heritage is a consequence of the various degradation phenomena that affect all materials, natural or artificial, used in building construction. Degradation can be defined as that process that involves harmful modifications of building materials, causing deterioration of their chemical-physical and structural characteristics. The visible effects of this phenomenon vary depending on the material, and they are products of the action caused by different weather conditions and physical, chemical, and biological factors that induce a progressive dissolution of the mineral matrix of the material used in the construction of the building. The safety of existing buildings, therefore, is a problem of fundamental importance, both for the high seismic vulnerability and for the historical-architectural-cultural-environmental value of the existing building heritage.

**Figure 1:**
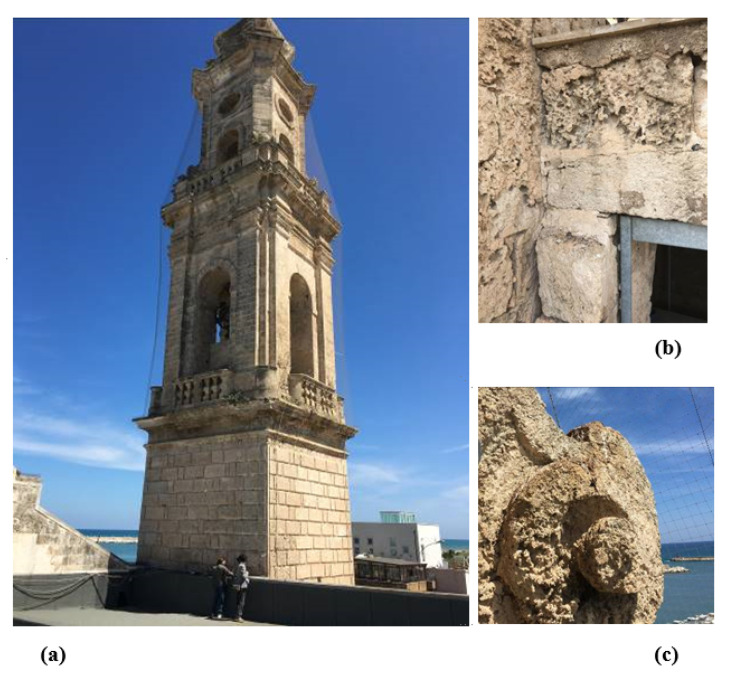
Belltower of Santa Maria di Loreto. Mola di Bari. (**a**) General view of the belltower. (**b**) Calcarenite stone superficially damaged by environmental effects near one of the access doors to the tower. (**c**) Ornamental element of calcarenite stone superficially damaged by environmental effects.

Figure 1 shows the superficial damage observed in the belltower of Santa María di Loreto in Mola di Bari. This belltower has a direct exposition to the sea wind loads, and these aggressive environmental effects have produced superficial damages on the structural calcarenite stone. This structure is a suitable example of the possible application of the proposed method for the consolidation of the superficial damage observed.

In this article, the effectiveness of the bioconsolidation technique applied to degraded structural materials has been illustrated as a new method of consolidation and conservation of the existing building heritage. The efficacy of the technique has been evaluated by an experimental campaign carried out with non-destructive diagnostic tests, static destructive mechanical tests, and microstructural analysis on a series of specimens before and after the use of bioconsolidants.

In this context, a promising method to limit the degradation of the stone can be found through the activation of the precipitation of calcium carbonate minerals with the use of bacteria. In recent years this method has been designed to face and resolve different engineering problems, including the recovery of stone and concrete elements. In-depth studies have been conducted in order to analyze the use of different bacteria and fungi to identify favorable factors for activating the calcium carbonate precipitation process [1,2]. The authors of [1] show that processes of microbial-induced calcium carbonate precipitation can be used for the production of multifunctional materials but also for permeability and strengthening the loosely cemented layers, consolidation of particles. A complete description of sustainable conservation and, in particular, of biotechnology applied to the preservation of cultural heritage is shown in the work of [3]. The use of microorganisms offers both opportunities and challenges and the potential of microorganism’s pro- and against-deterioration of cultural materials (e.g., stones, metals, graphic documents, textiles, paintings) [3,4,5].

Furthermore, the carbonate medium influences the sol-gel processes, and the perturbation of the sol-gel process due to a carbonate medium is studied in the work of [6,7]. The consequences of this effect on the consolidation quality are investigated, and strategies to minimize such problems are proposed.

Calcium carbonate bacterially induced precipitation was selected for its increased capability for biomineralization on marble, under different concentrations and temperature conditions, soft limestone stone of historic monument [8,9,10,11,12]. In the work of [13], identification and characterization of the bacterial and archaeal populations in a limestone monument were conducted by deepening the importance of the ecosystem on the colonization by different macrobiotic groups. The enormous impact of the bioconsolidation methodology based on the application of a sterile nutritive solution on the indigenous bacteria present in stone and plaster at the Maya archeological site of Copan is presented in the work of [14] with a detailed characterization of the bacterial population evolution that reveals the bioconsolidation treatment that induced a significant increase in beneficial indigenous carbonatogenic bacteria and a concomitant suppression of potentially damaging acidobacteria.

The successful application in the practice of microbiologically induced carbonate precipitation for limestone conservation in a wide range of environmental conditions is investigated in the work of [15]. In particular, a selection of microorganisms that are most suited for biodeposition at temperatures relevant for practice was studied. The influence of temperature on the performance of the biodeposition treatment with different ureolytic microorganisms was evaluated, both on the consolidative and protective effects of the treatment. Considerable research has focused on the biodeterioration and biodegradation of stone works important to our cultural heritage, including ancient monuments, historic buildings, and artworks, and developing novel conservation methodologies based on calcite precipitation with the use of bacteria [16,17,18,19,20,21,22].

An aspect still being explored concerns the use of microbiological calcium carbonate precipitation to improve the compressive strength of construction material. Few searches are available on this topic, as shown in the work of [20]. The first results show that microbiological calcium carbonate precipitation induces a positive effect on compressive strength.

While the microbial colonization of the monuments has been well documented in many studies for the knowledge of the microbial metabolic processes that affect and deteriorate the monument stones, other mechanisms such as the ability to improve the mechanical properties of the stone with this technique are not yet clear.

## 2. Bioconsolidation: Microbial Contribution to the Conservation of the Existing Building Heritage

The process of bacterial carbonatogenesis consists of a process of bacterial biomineralization that emerged as an ecological methodology for the conservation of deteriorated monuments, in particular those consisting of carbonate rocks (such as limestone, marble, and travertine) composed mainly of calcium carbonate (CaCO_3_).

In nature, the production of calcium carbonate is mainly attributed to various physical and chemical processes. However, the process presented in this study regarding the biological formation of the ionic compound is linked to the intrinsic properties of some microbial communities and, therefore, of certain carbonatogenesis microorganisms, also called calcifying microbes [23]. Consequently, the carbonatogenesis process is the result of biochemical reactions between ions and compounds that under optimal conditions determine the precipitation of extracellular calcium carbonate (around the cell, determining the mineralization of bacterial cells) induced by the microorganism.

Microbiological production of calcium carbonate (calcite) can occur in two ways:

- In an autotrophic way;
- In a heterotrophic way.

The precipitation of autotrophic calcium carbonate is promoted by methanogenic non-methylotrophic, atoxic, and oxygenic bacteria. These bacteria use carbon dioxide (CO_2_) in both gaseous and liquid form from a variety of sources, including breathing and fermentation. The use of carbon dioxide in a calcium-rich environment promotes the precipitation of calcium carbonate [23].

The production of calcium carbonate from heterotrophic bacteria can be active or passive, depending on the different mechanisms [23,24,25]. Passive production makes use of two metabolic cycles, the nitrogen cycle and the sulfur cycle, and uses three routes [23,24,25,26,27]:

- The ammonification of amino acids under anaerobic conditions in the presence of organic matter and calcium;
- The dissimilar reduction in nitrates under anaerobic and microaerophilic conditions in the presence of organic matter, calcium, and nitrate;
- The hydrolysis of urea or uric acid in the presence of urease and in an environment rich in organic substances and calcium.

**Figure 2:**
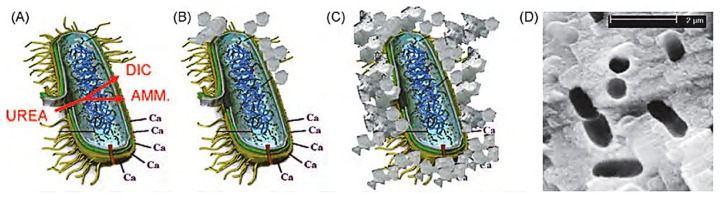
Simplified representation of events occurring during microbially induced carbonate precipitation. Calcium ions in the solution are attracted to the bacterial cell wall due to the negative charge of the latter. When urea is added to the bacteria, dissolved inorganic carbon (DIC) and ammonium (AMM) are released into the bacteria microenvironment (**A**). In the presence of calcium ions, this can cause local over-saturation and thus heterogeneous precipitation of calcium carbonate on the bacterial cell wall (**B**). Subsequently, the entire cell is encapsulated (**C**), limiting nutrient transfer, resulting in cell death. The image (**D**) shows the imprints of the bacterial cells involved in the precipitation of carbonate [28].

The active production, instead, does not depend on a sequence of biochemical reactions but is the result of the ion exchange through the cell membrane with a mechanism still little known [23,24,25]. In conclusion, it is important to stress that bacterial carbonatogenesis is governed by four key factors (Figure 2):

- Concentration of dissolved inorganic carbon (DIC);
- Calcium ion concentration;
- pH;
- Availability of nucleation sites and/or development of crystals for nucleation.

In order for the process to work, respecting the principle of physico-chemical compatibility, the degraded monument must be treated with a bacterial culture (nutritional solution) with characteristics very similar to those of the microorganism inherent in the rock, determining the consolidation through the formation of a cement (hybrid) of calcite and vaterite.

## 3. Selection of Stone Materials

### 3.1. Natural Stone Material: Novelda Stone

Among the natural building and ornamental materials of Spain, the limestone, called Novelda stone, occupies the most important position for its application as ornamental material [29,30,31] more commonly known by its trade name of Bateig stone. According to the petrographic classification of natural rocks, Bateig stone is an organogenic sedimentary rock [32] composed mainly of calcium carbonate (CaCO_3_). It is a fine-grained limestone rock [31,33] allochemical (mainly biosparite-biomycrite) very fossiliferous (biocalcarenite) with a compact and homogeneous structure [24,25,26,27,28,29,30,31,32,33,34,35,36] whose extraction, limited to the municipality of Elda (Alicante) [29,30,31], takes place from quarries, pit, open pit typical of the flat areas [33,37]. The extraction site [29] of each block strongly affects the exterior appearance of the stone. In fact, the Bateig stone available on the market takes on different names [38] due to the different shades as well as the different petrographic characteristics that distinguish each block of extracted stone material.

For the purposes of the experimentation, it was decided to work with different types of Bateig stone, in particular with the varieties: Blue, Beige Hydra, Crema Maroc, and Diamante. The aim is to understand which of these different types of natural stone will best respond to the subsequent bio-consolidating treatment.

### 3.2. Artificial Stone Material: Lime Mortar

Lime mortar is a composite artificial stone conglomerate formed, in fact, by a stone component or an aggregate of fine aggregates (sand) connected together by means of the adhesive component (plastic compound called binder paste), of inorganic nature, consisting of a mixture of water and binder (lime) [32,39]. The homogeneous mixture, thus obtained, of variable consistency, initially fluid and moldable, due to the set and hardening phenomena activated by the binder, hardens becoming solid and at the same time develops those significant mechanical characteristics and that degree of compactness typical of lithoid materials (the maturation process is the result of the complex chemical-physical transformations that occur when the binder interacts with water, hydrating) [32,39]. The final quality of the conglomerate depends not only on the nature of the single components [32,39] but, above all, on their proper use and dosage.

## 4. Determination of the Mechanical Characteristics of Untreated Specimens

The preliminary phase, necessary for the correct evaluation of the mechanical resistance of the specimens in stone material, natural and artificial, is the preparatory phase.

### 4.1. Preparation of the Specimens

Before performing the failure tests, the different prismatic Bateig stone specimens with standard dimensions 40 × 40 × 160 mm have been subjected to geometric regularity tests, as well as perpendicularity and flatness of the faces; in order to avoid that, during the test, undesired failures occur due to stress concentrations or tensile stresses generated by the eccentricity of the load.

Table 1 shows the set of specimens analyzed in the present work as well as the coding carried out in the study, indicating the results treated and not treated with the bioconsolidation process.

**Figure 3:**
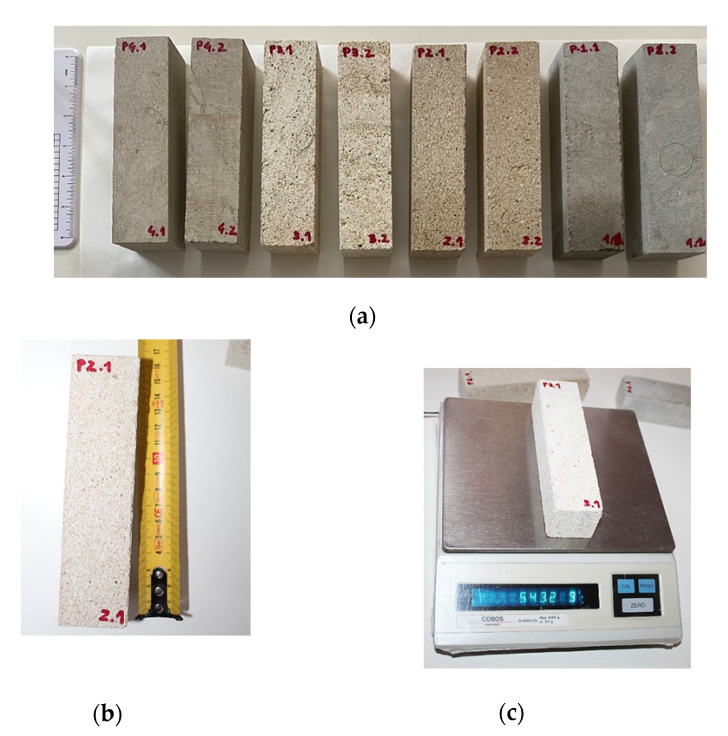
(**a**) Bateig stone specimens: Blue variety (P1.1/P1.2); Beige Hydra variety (P2.1/P2.2); Cream Maroc variety (P3.1/P3.2); Diamante variety (P4.1/P4.2). (**b**) Check of the geometric regularity and (**c**) sample weighing.

The phase of verification of geometric regularity was followed by the weighing phase of the Bateig stone specimens, each of which was named with a code, identifier, alphanumeric (Figure 3).

**Figure 4:**
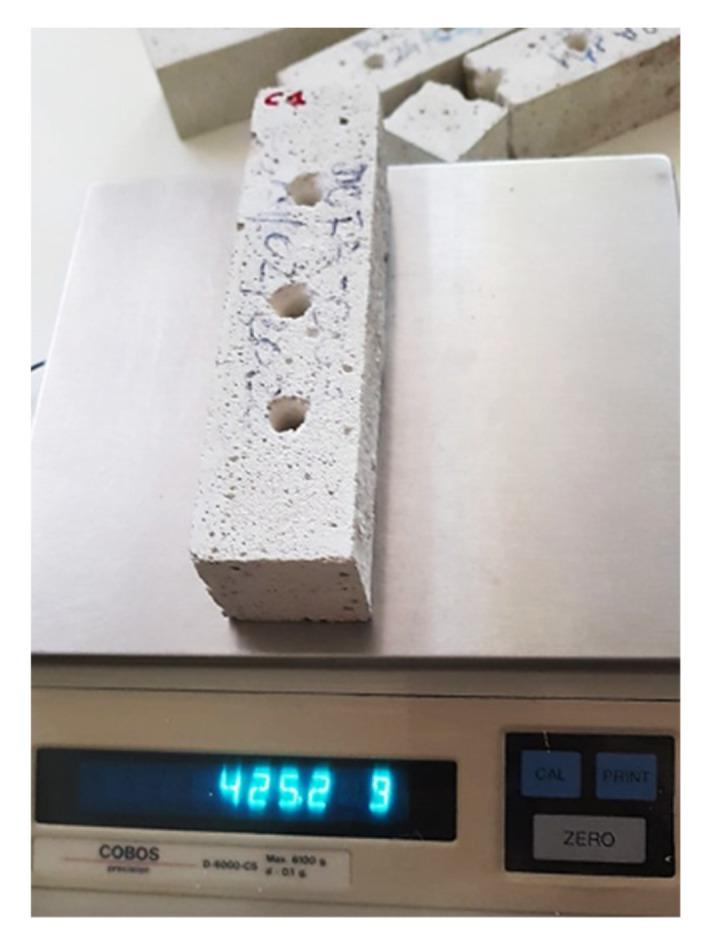
Weighing of the drilled sample in lime mortar.

As far as lime mortar specimens are concerned, unlike the previous Bateig stone specimens, they have been directly made in the laboratory in accordance with UNI EN 196-1:2016 “Cement test methods—Part 1: Determination of mechanical strength”. At the end of the complete drying period (10 days), the prismatic specimens in lime mortar (standard dimensions 40 × 40 × 160 mm) were weighed and finally drilled to simulate the deterioration, which is necessary to study the behavior and the mechanical capacity eventually acquired by the material following the subsequent application of the bioconsolidant (Figure 4).

### 4.2. Types of Tests Performed

Non-destructive diagnostic tests and static destructive mechanical tests were carried out, the latter aimed at determining the mechanical strength.

#### 4.2.1. Non-Destructive Diagnostic Test: Direct Ultrasonic Test

**Figure 5:**
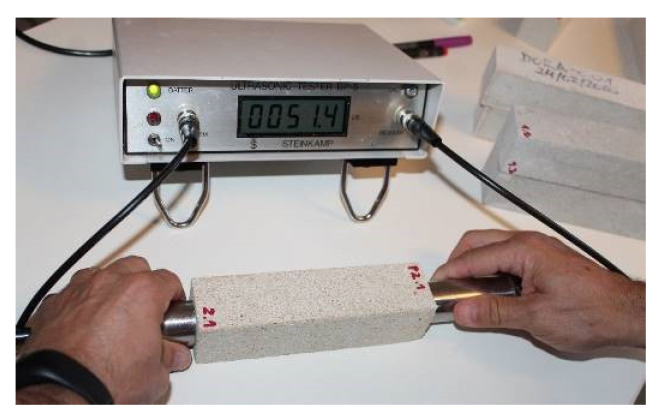
Sample P2.1 (Bateig Beige Hydra) subjected to direct ultrasonic testing.

The lime mortar specimens, as well as the various Bateig stone specimens, were subjected to the ultrasonic test [40]. The ultrasonic tester (Steinkamp BP-5 model) was used to measure the transit time, i.e., the time taken by the ultrasonic pulse to travel the distance from the emitting probe to the receiving probe, passing through the specimen under study. The ultrasonic measurements, preliminary to the evaluation of the speed of propagation of the impulses, the ultrasonic measurements were performed by adopting a direct approach, simpler and more practical. In the direct approach, the two cylindrical probes (containing the piezoelectric transducers made of titanized zircon lead) are arranged longitudinally on the two faces, opposite and parallel, of the material to be investigated [40] (Figure 5). During the execution of the test, constant pressure was exerted on the probes so that they adhere correctly to the surface of the material, which in turn must be flat and free from any residual particles or other materials.

#### 4.2.2. Static-Type Destructive Mechanical Tests: Flexural Strength Test and Compressive Strength Test

**Figure 6:**
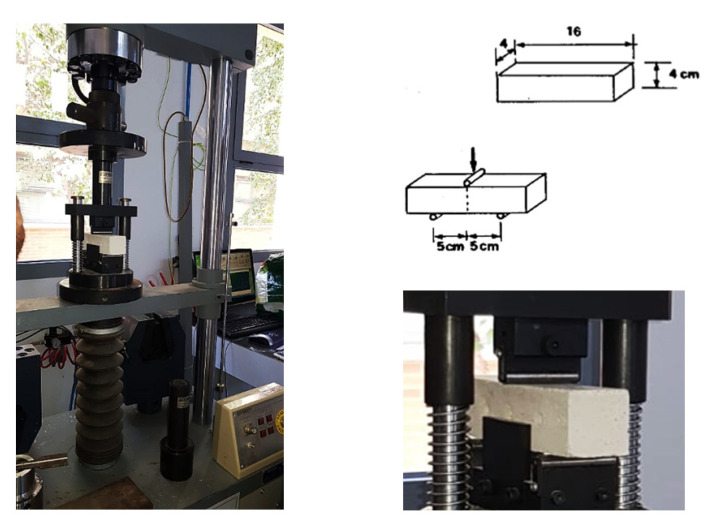
Multi-test machine equipped for the flexural strength test.

In compliance with the directives of the UNI EN 196-1:2016 standard, the flexural strength tests, and the compressive strength tests were performed by means of a multi-test press (Figure 6) equipped with the appropriate central loading body: two support rollers (100 mm apart) and the central loading roller for the flexural strength test; the lower support plate (surface equal to 40 × 40 mm) and the upper loading plate for the compressive strength test. The test methods are identical. When the circuit is closed, the specimen is placed in contact with the roller or the loading plate, which will exert, respectively, their gradual action until the material breaks. Only the compression strength tests are performed on the resulting halves obtained from the previous flexural test; they are, therefore, semi-prismatic specimens.

### 4.3. Experimental Results

#### 4.3.1. Direct Ultrasonic Test

Table 2 and Table 3 show the direct ultrasonic test values obtained for both stone materials included in this study. The speed of impulse propagation (V) was determined through the following relationship:

(1) $$V=\frac{L}{T}\;\left[\mathrm{km}/s\right]$$

where L = 160 mm, coincides with the length of the prismatic specimens and represents the distance traveled by the ultrasonic wave; the parameter T describes the time taken by the impulse to cross the entire length of the prismatic specimen.

#### 4.3.2. Flexural Strength Test

The values obtained from the flexural strength tests (F_f_) carried out on the different Bateig stone specimens are reported in Table 4. The flexural strength ($R_{f}$), also known as modulus of rupture, was obtained from the well-known formula of the linear elastic regime:

(2) $$R_{f}=\frac{1,5\times {\;F}_{f}\times \;l}{b^{3}}\left[\mathrm{MPa}\right]$$

where l = 100 mm represents the distance between the supports; b = 40 mm coincides with the side of the square prism section; finally, F_f_ is the maximum value of the collapse load measured during the test.

#### 4.3.3. Compressive Strength Test

Finally, the results of the compressive strength test performed on Bateig stone samples are reported in Table 5. The compressive strength ($R_{c}$) is obtained from the equation:

(3) $$R_{c}=\frac{F_{c}}{A_{c}}\left[\mathrm{MPa}\right]$$

where $A_{c}$ represents the surface of the auxiliary plates or plates and is 1600 mm^2^ (40 × 40 mm), while $F_{c}$ is the maximum value of the load for which the collapse occurred.

For each sample, we have two values, obtained, respectively, from the two halves resulting from the flexural failure test.

## 5. Experimental Procedure: Determination of the Mechanical Characteristics of the Bioconsolidated Specimens

Once the bioconsolidation of the stone materials under study was carried out, a quantitative determination of the mechanical resistance of the specimens and their microstructural analysis using optical microscopy and scanning electron microscopy (SEM) was performed.

### 5.1. Bioconsolidation of the Specimens

The product used for the bioconsolidation of the specimens was developed by the research group of the University of Granada and kindly granted by the Spanish company KBYO Biological, holder of the patent for the Myxostone bioconsolidation system. It is a nutritional solution M3P [12]. The superficially applied treatment is able to activate, among the microbial community that inhabits the stone, the bacteria with the potential to induce the precipitation of calcium carbonate. The product manufacturing process involves a rigorous and complex process of processing and autoclaving performed at constant temperature and pressure in order to avoid contamination of the culture, ensuring the effectiveness and purity of the product used in bioconservation and restoration of cultural heritage, sculptural monuments, consolidation of ornamental stones, reconstruction and repair of cracks in stone materials.

The application of the nutritional solution activates the microbiota inherent in the stone material itself, promoting the growth of carbonatogenic bacteria, which provide surface consolidation, through the formation of a consistent coating of (natural) calcium carbonate cement with a thickness of 1–1.5 μm perfectly adherent to the surface of the material, and with a depth of consolidation, well rooted in the porous system of the material. The formation of natural cement (calcite and/or vaterite crystals) favors, therefore, the regeneration and natural consolidation that results in an increase in the mechanical strength of the treated material without causing significant changes in porosity or surface color variations [12,23,41,42,43].

#### Application of the Nutritional Solution

**Figure 7:**
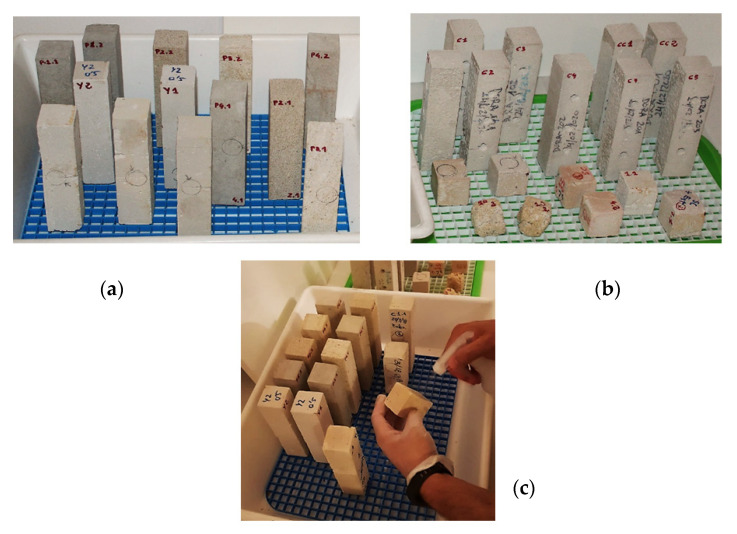
(**a**) Bateig stone specimens; (**b**) lime mortar specimens (**c**). Spray application of nutritional solution (**c**).

The effectiveness of this treatment has been tested directly on different Bateig stone and lime mortar samples. For each type of material, the specimens that lent themselves to the best treatment were identified based on the imperfections (small cracks, fissures), representative of their state of degradation. The nutritional solution was applied by spraying the specimens twice a day for seven consecutive days in an open environment, i.e., on the test bench where these were placed (Figure 7). In order to facilitate the activation of the autochthonous carbonatogenic microbiota inherent in the treated specimens, it was necessary to ensure a temperature of 20 °C inside the working environment.

### 5.2. Types of Tests Performed

In order for the process of bacterial carbonatogenesis to be triggered, determining the formation and growth of new calcite crystals responsible for the consolidation of the materials, it was necessary to wait a period of 16 days before proceeding with the evaluation of the mechanical characteristics by means of direct ultrasonic test and the bending and compression failure tests, the latter being preparatory to the subsequent microstructural analysis.

The same testing method equipment and parameters used for the determination of the mechanical characteristics of the unconsolidated specimens (see Section 4.2) were used for the bioconsolidated specimens. However, it is important to mention that bending and compression failure tests are a preparatory phase for the subsequent microstructural analysis by means of microscopy techniques.

### 5.3. Experimental Results

#### 5.3.1. Direct Ultrasonic Test

Table 6 and Table 7 show the values resulting from the ultrasonic test involving both Bateig stone samples and lime mortar samples, respectively. The speed of impulse propagation (V) was determined by (1) relationship.

#### 5.3.2. Flexural Strength Test

The values obtained from the flexural strength tests carried out on Bateig stone specimens (Table 8) and on lime mortar specimens (Table 9) are subsequently reported. From these, it was possible to obtain the flexural strength ($R_{f}$), through relationship (2) of the linear elastic regime:

#### 5.3.3. Compressive Strength Test

Finally, the results of the compressive strength tests carried out on Bateig stone specimens (Table 10) and on lime mortar specimens (Table 11) are reported. The compressive strength ($R_{c}$) is obtained from Equation (3).

## 6. Qualitative Analysis by SEM and Optical Microphotography

### 6.1. Microscopic Techniques for Microstructural Analysis of Samples Treated with MYXOSTONE M3P

The properties of a material do not depend only on its chemical composition; valuable information can also be obtained from the study of its microstructure, which, in this case, is referred to as microstructural analysis [44]. This analytical approach allows studying the microstructure of the material and the microstructural constituents both at the micrometric and sub-micrometric levels. The microstructural analysis can be performed with different techniques, among which the most important ones are the microscopy techniques [44] able to solve and enlarge objects that are not visible to the human eye (resolution power of the human eye is about 0.1 mm). For the microstructural analysis of the samples considered here, the optical microscopy technique and the scanning electron microscopy technique were chosen.

#### Optical Microscopy

**Figure 8:**
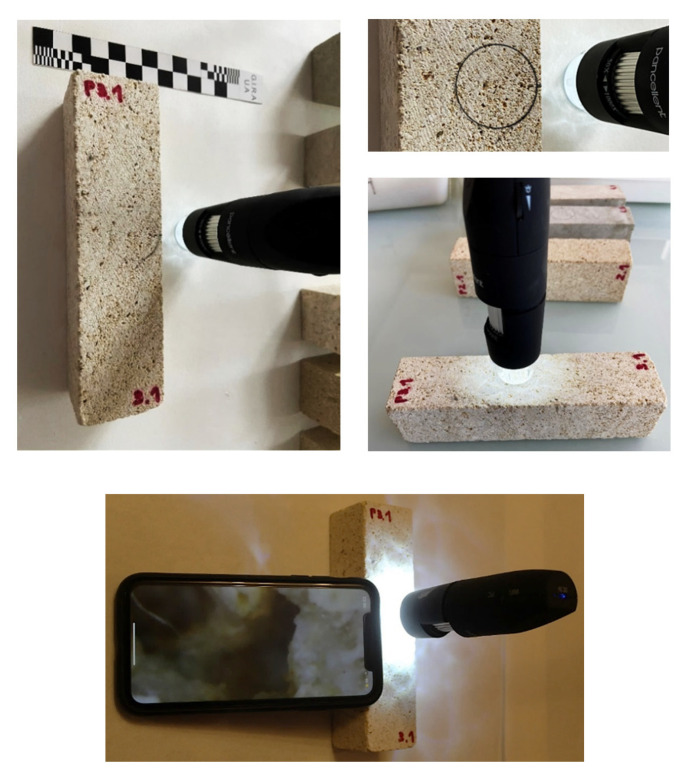
Specimen P3.1 (Bateig Crema Maroc) subjected to microscopic analysis using the Pancellent wireless digital optical microscope.

The instrument adopted for the microscopic analysis of the samples [44] is the wireless digital optical microscope model Pancellent 1080P (image and video acquisition 1920 × 1080P) that allows obtaining magnifications ranging from 50 × (50 μm) to 1000 × (0.2 μm). It is equipment that works in reflection, and it uses electromagnetic radiation in the visible field as a source to build and return the final, flat image of the material under investigation. Before proceeding with the microscopic analysis, a circle of diameter equal to the diameter of the lamp has been drawn on the surface of the samples in order to delimit the area under investigation (Figure 8).

### 6.2. Experimental Results: Optical Photomicrographs

**Figure 9:**
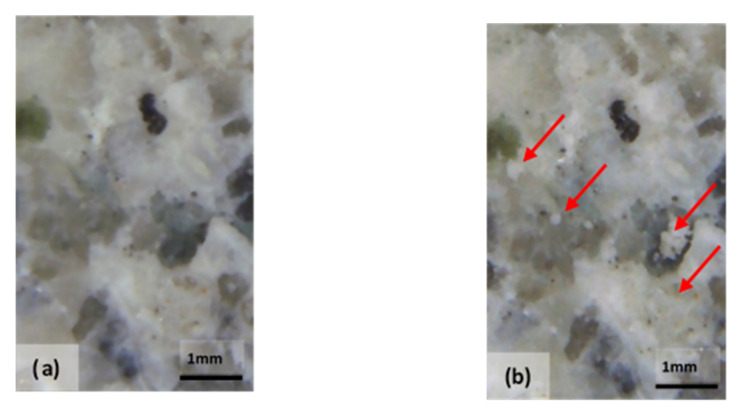
Optical photomicrograph: area of the specimen made of Bateig Blue variety stone (**a**) not treated with the bioconsolidant MYXOSTONE M3P (**b**); area of the same specimen subjected to the bioconsolidation. The arrows indicate the formation of calcite crystals four weeks after the start of treatment (**b**).

The effectiveness of the bioconsolidation treatment performed with the M3P nutritional solution inoculated with a *Myxococcus xanthus* culture showed its effects with the formation of new calcium carbonate crystals resulting from the extracellular precipitation of calcium carbonate, already four weeks after the application of the product (Figure 9).

### 6.3. Scanning Electron Microscopy

The SEM technique is performed on the fragments of the treated specimens previously subjected to breakage. This technique uses SEM to obtain very high-resolution images. The impact of the electron beam, used as a source, on the surface of the investigated sample generates different signals (secondary electrons, backscattered electrons, characteristic X-rays), which, suitably amplified, are used to modulate the intensity of the spot on the video screen obtaining the image of the sample area [44].

**Figure 10:**
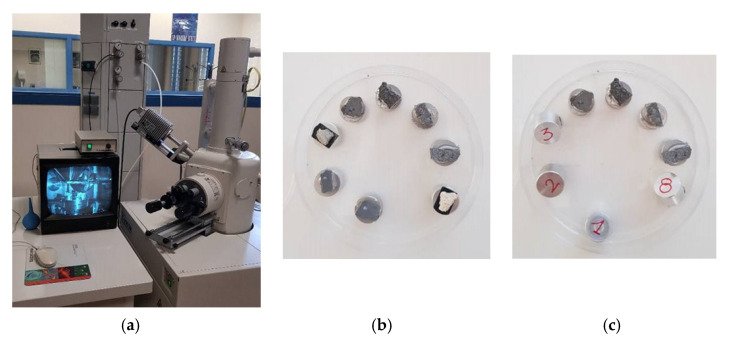
(**a**) S-3000N scanning electron microscope model Hitachi. (**b**,**c**) Samples mounted on the holder to be subjected to microstructural analysis.

For the purpose of the tests, the fragments of the specimens under study were subjected to microstructural analysis with a Hitachi model S-3000N scanning electron microscope (Hitachi, Tokyo, Japan), which allows magnifications ranging from 15× to 300,000×. Equipped with a Bruker brand XFlash 3001 X-ray detector (Bruker, Berlin, Germany) for EDS or EDX microanalysis and for chemical image processing (mapping), this device is an advanced system capable of working both at high vacuum (the ability of the detectors present in SEM to collect the generated signals, backscattered electrons, secondary electrons, and X-rays, is considerably increased) and in variable pressure from 1 to 270 Pa, for the observation of non-conductive samples without the absolute need to cover them with conductive material (Figure 10).

### 6.4. Experimental Results: SEM Photomicrograph

Table 12 reports the results obtained from the microscopic analysis performed with the scanning electron microscope of the various bioconsolidated samples. To highlight the results obtained, SEM micrographs of the same non-bioconsolidated materials have been compared.

## 7. Interpretation of the Results

### 7.1. Direct Ultrasonic Test: Pulse Propagation Speed

From the comparison of the results obtained (Table 13 and Table 14), it has been observed an increase in the speed of impulse propagation for both types of test specimens. This increase, although in some cases not very significant, was determined by the bioconsolidation treatment performed. In fact, the higher values of the propagation speed are a consequence of the shorter time taken by the impulse generated by the emitting probe to cross the entire prismatic sample until its capture by the receiving probe. In other words, the precipitation of calcium carbonate induced by carbonatogenic microorganisms (whose growth is favored by the microbiota inherent in the material itself activated by the nutritional solution) promotes the consolidation of the capillary pore system eliminating any inhomogeneity and/or defect in the material.

The knowledge of the propagation speed of ultrasonic waves is important since this parameter is closely related to the dynamic modulus of elasticity of the material, in turn, correlated with the compressive strength.

### 7.2. Flexural Strength

From the comparison of the results obtained (Table 15), an increase in flexural strength (highlighted in bold) was observed only for some Bateig stone specimens, in particular for the Diamante variety and the Blue variety; in the remaining cases, there was rather a decrease in the mechanical characteristics. This diversity in the resulting values is probably dictated by the structure of the capillary pore system. The Bateig Diamante and Bateig Blue varieties are characterized by a pore system whose diameter is smaller and therefore responds better to treatment, consolidating more effectively.

### 7.3. Compressive Strength

In addition, in this case, from the comparison of the results obtained (Table 16), an increase in compressive strength was observed only for some Bateig stone specimens, again for the Diamante variety and the Blue variety; in the remaining cases, there was rather a decrease in the mechanical characteristics. This condition can be justified by the fact that the compressive strength tests were performed on the resulting halves and, therefore, on the prism seeds obtained from the bending failure test. Therefore, the specimens showing an increase in the compressive failure strength correspond to the same specimens for which an increase in flexural strength was found.

### 7.4. Microstructural Analysis

**Figure 11:**
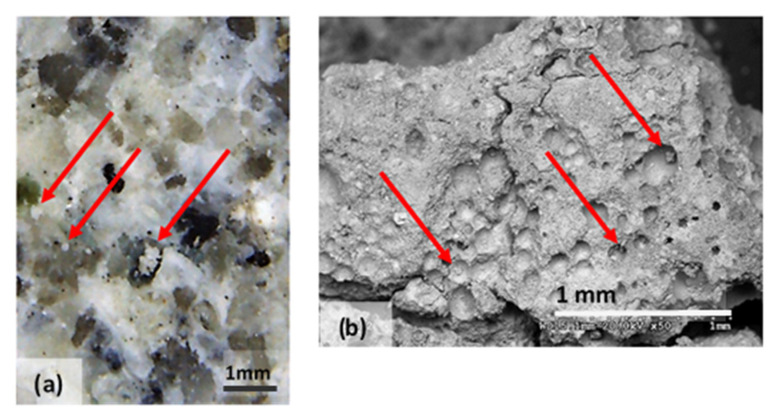
(**a**) Bioconsolidation of structural materials: in the optical microphotography of Bateig Blue (**b**) SEM microphotography, in backscattered electrons, of calcium mortar. It is possible to observe the formation of calcium crystals.

The microstructural analysis has demonstrated, concretely, the effectiveness of the treatment of bioconsolidation tested. In fact, four weeks after the application of the M3P nutritional solution inoculated with a Myxococcus xanthus culture, the formation of new calcium carbonate crystals promoting the structural consolidation of the materials under examination was observed by means of the optical and SEM (Figure 11).

## 8. Conclusions

From the results of the experimental study previously described, it is clear that the effect of the addition of a nutrition solution MYXOSTONE M3P for some varieties of Bateig and lime mortar produces a significant improvement, in quantitative terms, of the mechanical characteristics, meeting the expectations of the project. Therefore, according to what has been observed, bioconsolidation by precipitation of calcium carbonate induced by carbonatogenic microorganisms can be considered a valid alternative solution to traditional techniques (*scuci-cuci*, compensation; just to name a few) of consolidation and conservation of the existing building heritage.

Also, the economic aspect must be considered, indirectly linked to the environmental one. From what has been observed in this study, minimum quantities of products are required to achieve an effective improvement in material performance. This could certainly translate into a reduction in the overall costs of recovering the natural and/or artificial materials used in building construction.

Finally, in addition to an improvement in the mechanical performance, an increase in the durability and, therefore, the sustainability of the building material is clearly obtained.

For all said above, it is worth getting used to the idea that this highly selective solution, ecological and not harmful to human health, can become a well-established methodology in the field of recovery and conservation of existing building heritage.

## Figures and Tables

**Table 1:** Specimens analyzed for this research.

Material	Code	Untreated Specimen	Code Designation **	Treated Specimens	Code Designation **
Diamante Stone	P4	2	P4.X.UT	2	P4.X.T
Crema Maroc Stone	P3	2	P3.X.UT	2	P3.X.T
Beige Hydra Stone	P2	2	P2.X.UT	2	P2.X.T
Blue Stone	P1	2	P1.X.UT	2	P1.X.T
Lime Mortar	C	3	C.X.UT	3	C.X.T
Lime Mortar Control ***	CC	2	C.X.UT	-	-

** X is the number of the specimen (1, 2, 3). *** Untreated specimen to control the characteristics at the same fabrication age of treated specimens.

**Table 2:** Direct ultrasonic testing of unconsolidated Bateig stone specimens (mean values).

Bateig Stone (40 × 40 × 160 mm)	L (mm)	T (ms)	T (s)	V (km/s)
P4-Diamante	160	53.1	0.0531	3.013 × 10^−3^
P3-Crema Maroc	160	49.8	0.0498	3.213 × 10^−3^
P2-Beige Hydra	160	51.4	0.0514	3.113 × 10^−3^
P1-Blue	160	47.4	0.0474	3.376 × 10^−3^

**Table 3:** Direct ultrasonic testing of unconsolidated lime mortar specimens (mean values).

Lime Mortar (40 × 40 × 160 mm)	L (mm)	T (ms)	T (s)	V (mm/s)	V (km/s)
C	160	75	0.075	2134	2.134 × 10^−3^
CC ***	160	72.15	0.0725	2171.5	2.172 10^−3^

*** Untreated specimen to control the characteristics at the same fabrication age of treated specimens.

**Table 4:** Flexural strength test: unconsolidated Bateig stone specimens (mean values).

Bateig Stone (40 × 40 × 160 mm)	F_f_ (N)	R_f_ (N/mm^2^)
P4-Diamante	3130	7.34
P3-Crema Maroc	3380	7.92
P2-Beige Hydra	4850	11.37
P1-Blue	4880	11.44

**Table 5:** Compressive strength test: unconsolidated Bateig stone specimen (mean values).

Bateig Stone	F_c_ (N)	R_c_ (N/mm^2^)
P4-Diamante	52,100	32.57
P3-Crema Maroc	46,750	29.22
P2-Beige Hydra	87,250	54.54
P1-Blue	62,033.33	38.77

**Table 6:** Direct ultrasonic testing of Bateig stone specimens treated with MYXOSTONE M3P (mean values).

Bateig Stone (40 × 40 × 160 mm)	L (mm)	T (ms)	T (s)	V (km/s)
P4-Diamante	160	52.9	0.0529	3.025 × 10^−3^
P3-Crema Maroc	160	49.7	0.0497	3.219 × 10^−3^
P2-Beige Hydra	160	48.9	0.0489	3.272 × 10^−3^
P1-Blue	160	46.6	0.0466	3.433 × 10^−3^

**Table 7:** Results for direct ultrasonic testing of lime mortar specimens treated with MYXOSTONE M3P (mean values).

Lime Mortar (40 × 40 × 160 mm)	L (mm)	T (ms)	T (s)	V (mm/s)	V (km/s)
C	160	71.4	0.0714	2244.6	2.244 × 10^−3^
CC ***	160	71	0.07125	2213.5	2.213 × 10^−3^

*** Untreated specimen to control the characteristics at the same fabrication age of treated specimens.

**Table 8:** Flexural strength test: Bateig stone specimens treated with MYXOSTONE M3P (mean values).

Bateig Stone (40 × 40 × 160 mm)	F_f_ (N)	R_f_ (N/mm^2^)
P4-Diamante	6040	14.16
P3-Crema Maroc	3120	7.31
P2-Beige Hydra	3060	7.17
P1-Blue	5620	13.17

**Table 9:** Flexural strength test: lime mortar specimens treated with MYXOSTONE M3P (mean values).

Lime Mortar (40 × 40 × 160 mm)	F_f_ (N)	R_f_ (N/mm^2^)
C1	1066.67	2.5
CC **	1205	2.81

** Untreated specimen to control the characteristics at the same fabrication age of treated specimens.

**Table 10:** Compressive strength test: Bateig stone specimens treated with MYXOSTONE M3P (mean values).

Bateig Stone	F_c_ (N)	R_c_ (N/mm^2^)
P4-Diamante	88,215	55.14
P3-Crema Maroc	42,515	26.57
P2-Beige Hydra	45,000	28.125
P1-Blue	82,240	51.4

**Table 11:** Compressive strength test: lime mortar specimens treated with MYXOSTONE M3P. (mean values).

Lime Mortar	F_c_ (N)	R_c_ (N/mm^2^)
C1	9938.33	6.21
CC **	11,095	6.94

** Untreated specimen to control the characteristics at the same fabrication age of treated specimens.

**Table 12:** Results obtained from microscopic analysis. The red circles indicate the bioconsolidated areas.

**Sample**	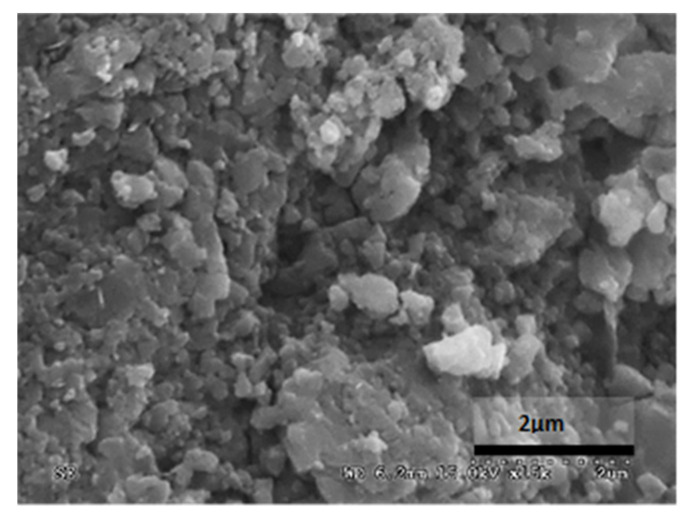
Blue Bateig Stone not bioconsolidated	
Conductive coating	
Iridium (Ir)	
SEM microphotography in secondary electrons	
Enlargement: X 15k = 15,000 X Depth of field: 2 μm	
**Sample**	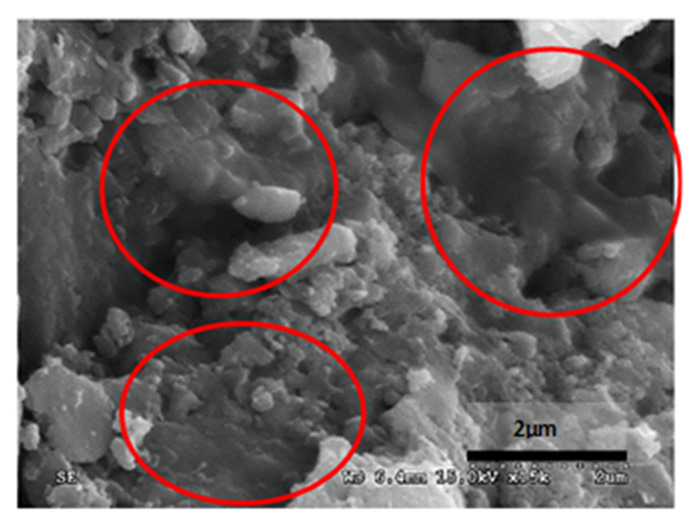

Bioconsolidated Blue Bateig Stone	
Conductive coating	
Iridium (Ir)	
Microfotografia SEM in secondary electrons	
Enlargement: X 15k = 15,000 X Depth of field: 2μm	
**Sample**	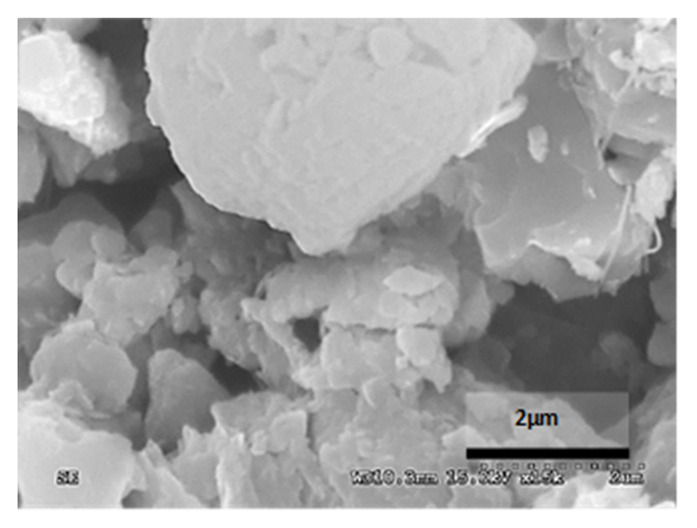
Maroc Cream Bateig Stone not bioconsolidated	
Conductive coating	
Iridium (Ir)	
SEM microphotography in secondary electrons	
Enlargement: X 15k = 15,000 X Depth of field: 2 μm	
**Sample**	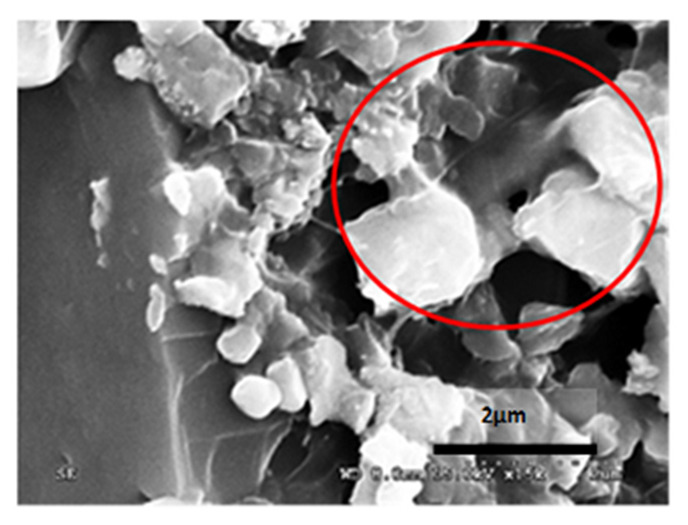
Bioconsolidated Bateig Stone Maroc Cream	
Conductive coating	
Iridium (Ir)	
SEM microphotography in secondary electrons	
Enlargement: X 15k = 15,000 X Depth of field: 2 μm	
**Sample**	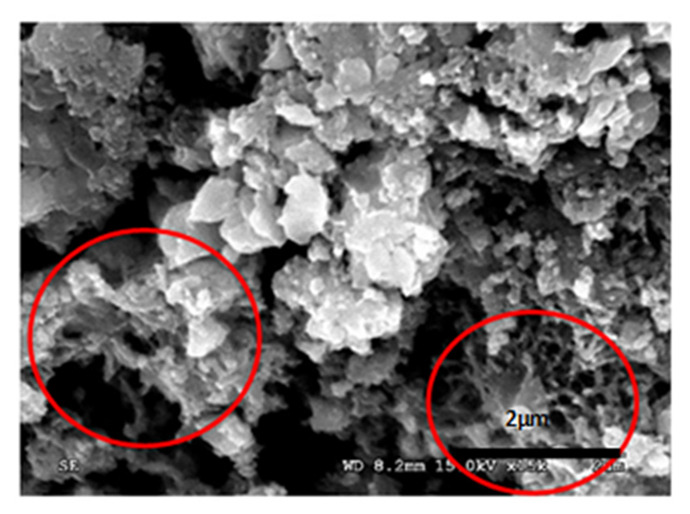
Lime mortar not bioconsolidated	
Conductive coating	
Iridium (Ir)	
SEM microphotography in secondary electrons	
Enlargement: X 15k = 15,000 X Depth of field: 2 μm	
**Sample**	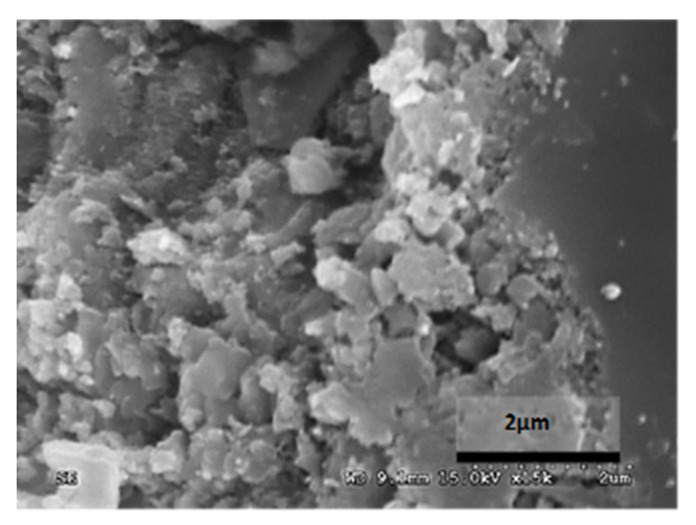
Bioconsolidated lime mortar	
Conductive coating	
Iridium (Ir)	
SEM microphotography in secondary electrons	
Enlargement: X 15k = 15,000 X Depth of field: 2 μm With 50× enlargement and a depth of field of 1 mm, the formation of calcium carbonate crystals can be observed.	
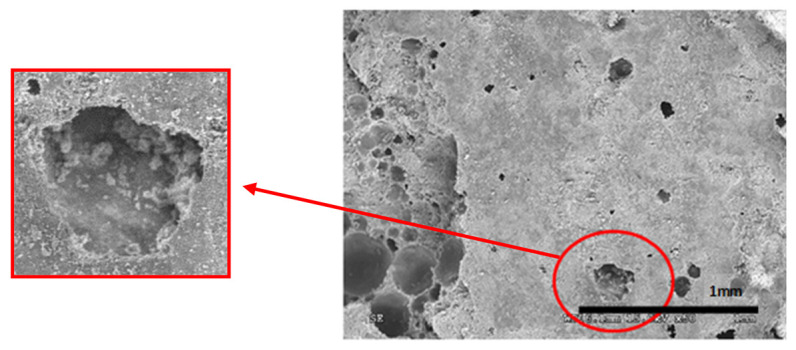	

**Table 13:** Comparison between the values of the impulse propagation rates before and after treatment of Bateig stone specimens (mean values).

Bateig Stone	L (mm)	Before Treatment	After Treatment
		V (m/s)	V (km/s)
P4-Diamante	160	3.013 × 10^−3^	3.025 × 10^−3^
P3-Crema Maroc	160	3.213 × 10^−3^	3.219 × 10^−3^
P2-Beige Hydra	160	3.113 × 10^−3^	3.272 × 10^−3^
P1-Blue	160	3.376 × 10^−3^	3.433 × 10^−3^

**Table 14:** Comparison between the values of the impulse propagation rates before and after treatment of lime mortar samples (mean values).

Lime Mortar	L (mm)	Before Treatment	After Treatment
		V (km/s)	V (km/s)
C	160	2.134 × 10^−3^	2.244 × 10^−3^
CC **	160	2.222 × 10^−3^	2.302 × 10^−3^ **

** Untreated specimen to control the characteristics at the same fabrication age of treated specimens.

**Table 15:** Comparison between the values of the flexural strengths before and after the treatment of Bateig stone specimens (mean values).

Bateig Stone	Before Treatment	After Treatment
	R_f_ (MPa)	R_f_ (MPa)
Diamante	7.34	14.16
Crema Maroc	7.92	7.31
Beige Hydra	11.37	7.17
Blue	11.44	13.17

**Table 16:** Comparison between the values of the compressive strengths before and after the treatment of Bateig stone specimens (mean values).

Bateig Stone	Before Treatment	After Treatment
	R_c_ (MPa)	R_c_ (MPa)
P4-Diamante	32.57	55.14
P3-Crema Maroc	29.22	26.57
P2-Beige Hydra	54.54	28.125
P1-Blue	38.77	51.4

## Data Availability

All the data is available within the manuscript.
